# The efficacy of hydrogen sulfide (H_2_S) tests for detecting microbial contamination in rooftop-harvested rainwater

**DOI:** 10.1007/s10661-023-11942-y

**Published:** 2023-11-01

**Authors:** Arthur Moses, Mónica D. Ramírez-Andreotta, Jean E.T. McLain, Victoria Obergh, Emma Rutin, Shana Sandhaus, Aminata P. Kilungo

**Affiliations:** 1 Department of Environmental Science, University of Arizona, 1177 E. Fourth St, Tucson, AZ 85721 USA; 2 Mel and Enid Zuckerman College of Public Health, Department of Community, Environment and Policy, University of Arizona, 1295 N. Martin Ave, Tucson, AZ 85721 USA; 3 Water Resources Research Center, University of Arizona, 350 N. Campbell Ave, Tucson, AZ 85719 USA

**Keywords:** Rainwater harvesting, Citizen science, Vulnerable populations, Total coliforms, *E. coli*, Sulfate-reducing bacteria

## Abstract

As climate change strains the world’s freshwater resources, access to safe and clean water becomes limited. The use of alternative water sources, such as rooftop-harvested rainwater, has become one mechanism to address freshwater scarcity in the American Southwest, particularly when it comes to home gardening. The University of Arizona’s Project Harvest, in partnership with the Sonora Environmental Research Institute, Inc., is a multi-year, co-created citizen science project aimed at increasing current understanding of harvested rainwater quality. Citizens in four Arizona, USA, communities (Hayden/Winkelman, Globe/Miami, Dewey-Humboldt, and Tucson) submitted harvested rainwater samples over 3 years. The harvested rainwater samples were then analyzed using IDEXX Colilert® for total coliforms and *E. coli* and using Hach PathoScreen™ test for sulfate-reducing bacteria (SRB). This study design allows for the validation of a low-cost, at-home alternative methodology for testing rainwater for bacteria that may indicate fecal contamination. In total, 226 samples were tested using both methodologies, revealing a positive correlation (*r*=0.245; *p*<0.002) between total coliform MPN and SRB MPN, but no discernable correlation between *E. coli* MPN and SRB MPN. This work indicates a potential value of SRB testing for harvested rainwater if cost, laboratory access, and fecal contamination are of concern.

## Introduction

### Rainwater harvesting and sulfate-reducing bacteria

In the twenty-first century, freshwater scarcity has continued to be a concern worldwide. It is estimated that approximately 4 billion people contend with severe water scarcity for at least 1 month out of the year (Mekonnen & Hoekstra, 2016). To combat water scarcity, a growing number of people have taken to rainwater harvesting in the American Southwest. While the harvested rainwater can be a valuable tool for combating water scarcity, pathways do exist for microorganisms to enter harvesting devices, which can be of concern if the water is being utilized for purposes such as irrigating edible food gardens or even as a potable source. Testing for indicator organisms, or organisms whose presence indicates a potential for pathogen presence, is one mechanism to assess biological contamination of water sources.

Coliform bacteria, often described in water quality monitoring as total coliforms (TC), are a group of generally harmless bacteria found in both the environment and the gastrointestinal tracts of humans and animals (Washington State Division of Environmental Public Health, 2016). *Escherichia coli* (*E. coli*) are a specific type of coliform known as a fecal coliform. *E. coli* are more common in the gastrointestinal tract than in the environment and they can be pathogenic (Washington State Division of Environmental Public Health, 2016). The presence of *Escherichia coli* (*E. coli*) and total coliforms (TC) are standards for assessing microbial water quality. However, alternative indicator organisms, such as sulfate-reducing bacteria (SRB), have also been employed to assess microbial water quality in various environments. The potential for warm-blooded animals to shed SRB is what allows the organisms to serve as a potential indicator organism for water contamination (Gupta et al., 2008). In addition, the ease of use and lower cost of testing for SRB when compared to traditional fecal coliforms has made it a popular choice where water quality is of concern (Sobsey & Pfaender, 2002). Our study assessed the reliability of using SRB as an at-home indicator organism for rainwater quality evaluation, and to provide tools for homeowners to assess their rainwater quality, especially those who use harvested rainwater to irrigate their produce.

SRB are prokaryotic microbes that help to facilitate nature’s sulfur cycle. In anaerobic environments, SRB utilize sulfate as a terminal electron acceptor, producing sulfide products, usually in the form of hydrogen sulfide (H_2_S). There are more than 220 known species of SRB, creating a plethora of microorganisms which could create a positive result using the SRB testing method (Barton & Fauque, 2009). SRB live in a wide range of environments, including oceanic waters and sediments, freshwater, brackish swamps, hydrothermal vents, hot springs, and deep subsurface soils (Fishbain et al., 2003). Like other residential water sources, rainwater harvesting devices are generally closed to the outside environment to reduce contamination. However, rainwater harvesting systems in Arizona, USA, are particularly hospitable to SRB, due to ambient temperatures (from a low of 17.5°C (290.65K) to a high of 30.1°C (303.25K)) which are within the 28°C (301.15K) to 30°C (303.15K) optimal growth range for SRB (U.S. National Weather Service, 2020; Virpiranta et al., 2019).

### About Project Harvest

The University of Arizona’s Project Harvest (PH), in partnership with Sonora Environmental Research Institute, Inc. (SERI), was designed as a co-created citizen science (CS) project focused on evaluating potential microbiological, organic, and inorganic pollutants in harvested rainwater, as well as in irrigated soil and grown plants. An integral part of Project Harvest involves training homeowners to test, and interpret testing results of, their harvested rainwater samples. This work describes efforts to determine if easy-to-use-and-interpret, low-cost SRB tests, such as Hach’s PathoScreen™ field test kits, are a viable alternative for at-home testing of microbial quality of harvested rainwater.

## Materials and methods

### Community recruitment and training

Recruitment for the project occurred throughout four Arizona, USA, communities: Dewey-Humboldt, Globe/Miami, Hayden/Winkelman, and Tucson. These communities were selected based on several critical factors, including their proximity to Toxic Release Inventory (TRI) sites and National Priorities List (NPL) sites, the research interest and/or concern expressed by community members, and previously established relationships between the PI and community leadership and members (Davis et al., 2020). Project Harvest employed a *promotoras* de salud (community health worker) model to better facilitate communication with partnered communities. *Promotoras* assisted in primary duties such as sample collection, recruitment, and training of participants. Participants were consented under the University of Arizona Institutional Review Board rule, ensuring the rights and welfare of human participants in research.

Before rainwater collection began, project participants in each community were supplied with kits which included all materials required to complete the sample collection and/or analysis (see https://projectharvest.arizona.edu/about#sampling-methodologies for details)

### Microbial rainwater assessment

In the first 2 years (2017–2019), PH participants were randomly assigned into one of two method analysis categories: Do-it-Yourself (DIY) and traditional lab (Lab). For year three (2019–2020), participants had the option of which method they preferred. One objective of both DIY and Lab participants was to determine the microbial quality of harvested rainwater as measured by the presence of indicator organisms. DIY participants were provided Hach PathoScreen™ field test kits (HACH, Loveland, CO) to analyze their collected rainwater samples at home for the presence and activity of SRB. Participants were instructed to report the data back to the University research team. In contrast, samples collected by Lab participants were transported to the University of Arizona, where the project team analyzed them using IDEXX Colilert® (IDEXX Laboratories, Westbrook, ME) for *E. coli* and TC bacteria.

Initially, the project was designed with the intention for participants to complete both the Lab and DIY methodologies in year three (2019–2020) creating a direct one-to-one comparison for validation. However, participant and *promotora* feedback (details below) revealed participant fatigue, making it no longer feasible to have participants conduct both methods. Instead, between 2018 and 2020, we analyzed the submitted Lab samples using both the IDEXX Colilert® and the Hach PathoScreen™ methods in order to validate the use of a low-cost, at-home alternative methodology for testing harvested rainwater. Both Lab and DIY participants submitted samples during December and February for the winter season, and June and September for the monsoon season, encompassing the major precipitation periods in Arizona, USA.

### *E. coli* and total coliform rainwater quality assessment (Lab method)

The Lab microbial analyses consisted of collecting water from rainwater harvesting tanks in a sterile plastic 250-ml sample bottle. Lab participants were instructed to drop off samples at designated points in their respective communities for retrieval and analysis by the University team. To validate the SRB method for harvested rainwater samples, Lab samples were tested for both TC and *E. coli* using Colilert®, and for SRB using PathoScreen™. All microbiological testing was done in accordance with manufacturer guidelines (IDEXX Laboratories, n.d.; Hach., 2016). Colilert® results range between <1.0 MPN/100ml (lower limit of detection (LOD)) and >2419.6 MPN/100ml (upper limit of quantification (ULOQ)). Similarly, PathoScreen™ has a LOD of <1.1 MPN/100ml and an ULOQ of >8.0 MPN/100ml.

### SRB rainwater quality assessment (DIY method)

**Fig. 1:**
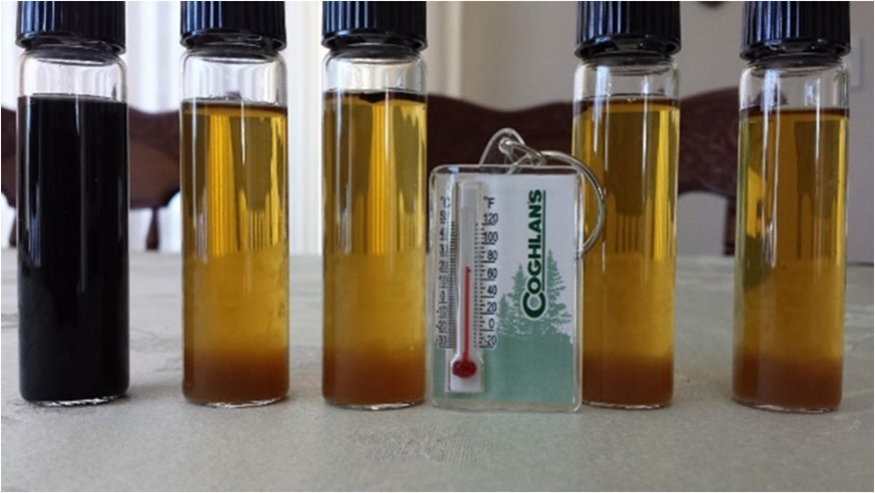
SRB vials indicating a positive sample via black precipitate (left)

**Fig. 2:**
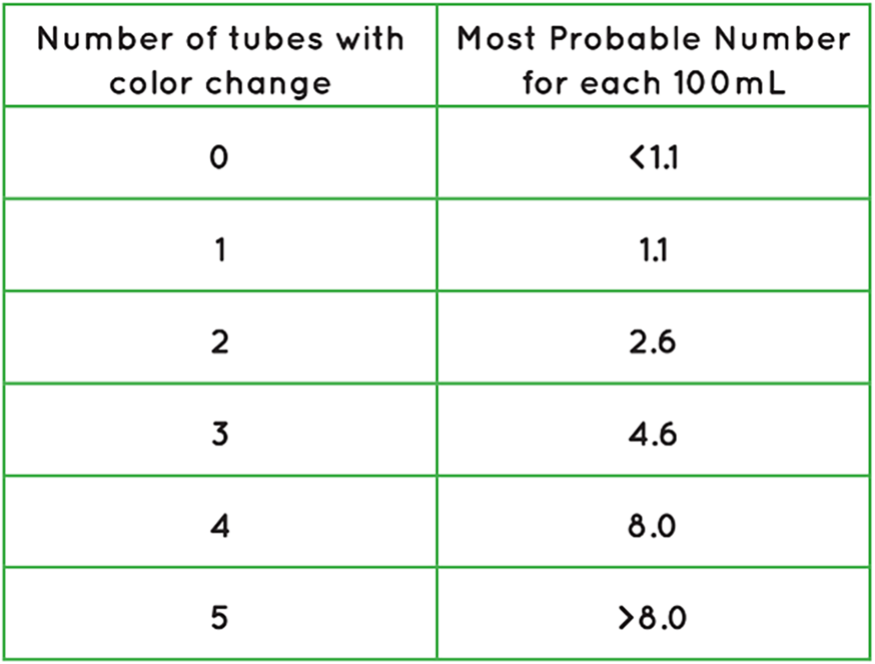
The SRB MPN chart distributed in handbooks to DIY participants for interpreting sample results

While completing the DIY method at home, participants collected rainwater samples in a sterile plastic 250-ml sample bottle. They then transferred 20 ml of their sample into five pre-marked sterile 25-ml glass vials, followed by addition of a powdered substrate supplied by the manufacturer and gently swirling to dissolve the substrate into the water sample. Participants then incubated the vials in a location at their home with a constant temperature between 25 and 30°C (298.15–303.15 K). Once a day, for 5 days, participants recorded the ambient temperature and any color change of water within each vial. Samples were “positive” if a change from the original amber color to black occurred, or if black precipitates were observed in any vials (indicative of sulfate reduction to form iron sulfides) (Fig. 1). If color change or black precipitates were observed, the positive vials were compared to a most probable number (MPN) chart (Fig. 2) to determine the concentration of SRB in the sample. Once the participant completed the experiment at home, they submitted their results, with the option to send vial pictures, to the Project Harvest research team via the Project Harvest website and journal entry portal, email, text message, or a physically mailed worksheet. Once the online portal system was established, we recognized that technological comfort and access may prove to be a barrier for returning results. Alternatively, we opted to receive DIY-tested SRB results through a paper worksheet, which was added as a submission method. While this did not allow us to get photos for validation, it improved results submissions.

### Participant interaction and preference

In addition to ongoing data sharing and participant contact, to better garner and understand participant sampling method preference (DIY or Lab) and rationale, we hosted “Open House” events towards the end of Year 2 (2018–2019). At these events, participants were prompted to sign up for the kit of their choice for the third sampling year (2019–2020). Participants who were not in attendance were given the option to be interviewed by phone, and the remaining participants were contacted through a text campaign, prompting the selection of which method, DIY or Lab, they would like for Year 3. Participants who did not respond with a kit preference were divided into two groups. Those who were present since the beginning of the study were automatically assigned a Lab kit. To ensure all participants a chance to experience both kit types, those who joined in Year 2 were assigned the opposite kit from the one they recently completed.

### Statistical analysis on water quality methods

For statistical comparison, microbial results below the LOD for both tests were recorded as half of the LOD (e.g. <1.0 MPN for Colilert® was recorded as 0.5 MPN), and the ULOQ was rounded to 2420.0 and 8.1 for Colilert® and PathoScreen™ respectively. Taking half the limit of detection is a standard practice for dealing with censored values in environmental monitoring; separately, the ULOQ decision was made to ensure all censored values were constant (Croghan & Egeghy, 2003). Results from both Colilert® and PathoScreen™ were recorded in Microsoft Excel (Seattle, WA, 2016 Version 16.0) spreadsheets. The results were then uploaded into R Studio software (Boston, MA, 2020 Version 3.6.3) for statistical processing.

The relationship between *E. coli* MPN and SRB MPN was measured using a Spearman rank correlation test as values above the ULOQ were not discretely known without a dilution series. Spearman’s serves as a non-parametric test for measuring the monotonic relationship between two variables. Presence/absence categories for both tests were also recorded, and the subsequent data was tested for association using Pearson’s chi-square test. Finally, a point-biserial correlation was conducted to determine the correlation between Colilert® MPN and SRB presence/absence.

## Results

### SRB vs coliform bacteria (Lab method)

**Fig. 3:**
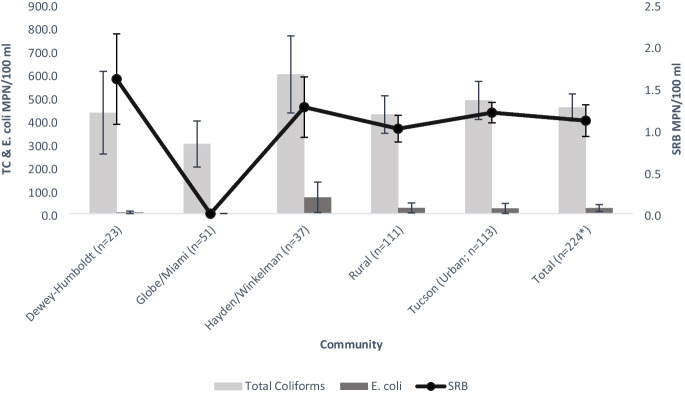
Harvested rainwater MPN values by community. A breakdown by community location and type of the average MPN values from the Lab participant–submitted samples. *Two samples were from Arizona Trail System and were not classified in any specific community

**Fig. 4:**
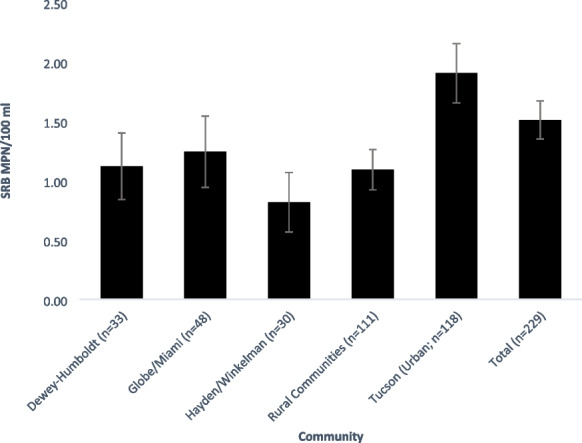
Community submitted harvested rainwater MPN values. A breakdown by community of the average MPN values from submitted samples

As previously stated, we modified our approach to validate the SRB method. In the second and third year, 2018–2020, a total of 226 samples were collected by Lab participants and submitted to the University of Arizona. These samples were tested for SRB and TC utilizing both DIY and Lab methodologies. The majority (*n*=200, 88.5%) of collected samples were negative for SRB, only 26 (11.5%) were positive (Table 1). The average MPN for all SRB samples was 1.1 MPN/100 ml (Fig. 3). Samples were tested for both TC and *E. coli* concentrations, then compared to SRB results. SRB MPN had a positive correlation (*r*=0.245, *p*<0.05) with TC MPN (Fig. 3). There was no discernable correlation with *E. coli* MPN and SRB MPN in harvested rainwater (Fig. 4).

**Table 1:** Bacteria presence/absence by community

Community	Total coliforms		*E. coli*		SRB	
	Present	Absent	Present	Absent	Present	Absent
Dewey-Humboldt (*n*=23)	13 (56.5%)	10 (43.5%)	2 (8.7%)	21 (91.3%)	6 (26.1%)	17 (73.9%)
Globe/Miami (*n*=51)	33 (64.7%)	18 (35.3%)	2 (3.9%)	49 (96.1%)	1 (2.0%)	50 (98.0%)
Hayden/Winkelman (*n*=37)	19 (51.4%)	18 (48.6%)	3 (8.1%)	34 (91.9%)	4 (10.8%)	33 (86.7%)
Tucson (*n*=113)	73 (64.6%)	40 (35.4%)	13 (11.5%)	10 (88.5%)	15 (13.3%)	98 (86.7%)
Arizona Trail System (*n*=2)	0 (0.0%)	2 (100.0%)	0 (0.0%)	2 (100.0%)	0 (0.0%)	2 (100.0%)
Total	138 (61.1%)	88 (38.9%)	20 (8.85%)	206 (91.2%)	26 (11.5%)	200 (88.5%)

A breakdown by community on the presence of coliform bacteria, *E. coli*, and SRB from the Lab participant’s submitted samples

After MPN comparisons, the SRB results were given a presence/absence comparison against TC and *E. coli* presence/absence (Tables 3 and 4). In 82 samples (36.3%), neither coliform bacteria nor SRB were detected. In 118 samples (52.2%), Colilert® detected coliform bacteria but no SRB were detected via the DIY method. In 5 samples (2.2%), Colilert® did not detect any coliform bacteria, and the DIY method did detect SRB. In 21 samples (9.3%), coliform bacteria and SRB were both detected (Table 2). This produced an approximate 45.6% agreement rate between TC and SRB for detecting water contamination. The majority, 184 samples (81.4%), were negative for both *E. coli* and SRB (Table 3). Conversely, only 4 samples (1.8%) were positive for both *E. coli* via Colilert® and SRB via PathoScreen™ (Table 4). Bacterial load did vary by seasonality, though the presence/absence of our target organisms was fairly consistent across the two rainy seasons (Tables 4 and 5). Overall, SRB presence/absence had a positive correlation (*r*=0.1428, *p*<0.05) with TC presence/absence. When tested against *E. coli* presence/absence, SRB presence/absence did not have a statistically significant correlation.

**Table 2:** Contingency table for total coliforms and SRB presence/absence

		Total Coliforms		Total
		Negative (−)	Positive (+)	
SRB	Negative (−)	82	118	200
	Positive (+)	5	21	26
Total		87	139	226

Contingency table demonstrating the binary values for the presence/absence of Total coliforms and SRB evaluations

**Table 3:** Contingency table for *E. coli* and SRB presence/absence

		*E. coli*		Total
		Negative (−)	Positive (+)	
SRB	Negative (−)	184	16	200
	Positive (+)	22	4	26
Total		206	20	226

Contingency table demonstrating the binary values for the presence/absence of *E. coli* and SRB evaluations

**Table 4:** Bacteria presence/absence by harvesting season

Season	Total Coliforms		*E. coli*		SRB	
	Present	Absent	Present	Absent	Present	Absent
Monsoon (*n*=90)	58 (64.4%)	32 (35.6%)	7 (7.8%)	83 (92.2%)	11 (12.2%)	79 (87.8%)
Winter (*n*=136)	81 (59.6%)	55 (40.4%)	13 (9.6%)	123 (90.4%)	15 (11.0%)	121 (89.0%)

A breakdown by season on the presence of coliform bacteria, *E. coli*, and SRB

**Table 5:** Harvested rainwater MPN values by harvesting season

Community	Descriptive statistic	Total coliforms MPN/100 ml	*E. coli* MPN/100 ml	SRB MPN/100 ml
Monsoon (*n*=90)	Mean	581.2	56.8	1.1
	Median	11.5	<1.1	<1.1
Winter (*n*=136)	Mean	367.8	2.3	1.1
	Median	7.5	<1.1	<1.1

A breakdown by collection season of the average and median MPN values from submitted samples

### Participant self-reported results

Between 2017 and 2020, DIY participants reported their at-home results for 229 SRB tests. The majority (*n*=182, 79.5%) of participant samples were negative for SRB, only 47 (20.5%) were positive (Table 6). The average MPN for all participant-submitted SRB samples was 1.51 MPN/100 ml (Fig. 4). The median for those samples was <1.1 MPN/100 ml, and the geometric mean was also <1.1 MPN/100 ml. SRB MPN varied with seasonality, though this was not significant (*p*>0.05) (Table 7).

**Table 6:** Community submitted SRB presence/absence by community

Community	SRB	
	Present	Absent
Dewey-Humboldt (*n*=33)	6 (18.2%)	27 (81.8%)
Globe/Miami (*n*=48)	7 (14.6%)	41 (85.4%)
Hayden/Winkelman (*n*=30)	2 (6.7%)	28 (93.3%)
Tucson (*n*=118)	32 (27.1%)	86 (72.9%)
Total (*n*=229)	47 (20.5%)	182 (79.5%)

A breakdown by community on the presence of coliform bacteria, *E. coli*, and SRB

**Table 7:** Community submitted harvested rainwater MPN values by harvesting season

Season	Descriptive statistic	SRB MPN/100 ml
Monsoon (*n*=111)	Mean	1.65
	Median	<1.1
Winter (*n*=118)	Mean	1.38
	Median	<1.1

A breakdown by collection season of the average and median MPN values from submitted samples

### Participant reported feedback

Participants were given the option to choose either the DIY or Lab method. Thirty-eight (60.3%) participants chose to complete the Lab method, 17 (27.0%) selected a DIY method, and 8 (12.7%) chose to complete both methods for Year 3. The remainder of (*n*=91) were assigned the Lab method.

## Discussion

### Sulfur, sulfate, and SRB in the home environment

While we did observe positive results, particularly between total coliforms and sulfate-reducing bacteria, there are other sources of sulfates in the environment. In Arizona, there are potential mechanisms in which sulfates may enter rainwater harvesting systems. As sulfates are the main electron acceptor for SRB in anaerobic environments, environments containing them provide a natural habitat for their growth (Phyo et al., 2020). There exist several pathways for potential sulfate contact with water sources in residential settings. In Arizona, mining is a dominant industry, with 380 active mines recorded in 2019 (Richardson et al., 2019). One common byproduct of the mining industry is acid rock drainage (ARD) in surface waters, which contains sulfuric acid (H_2_SO_4_), among other compounds (Dos Santos et al., 2016). Mine tailings piles also commonly contain sulfide compounds such as pyrite (FeS_2_), which upon exposure to a humid atmosphere can oxidize to ARD (Dos Santos et al., 2016; Lim et al., 2009). Tailings piles are present in the Project Harvest partner communities, where eolian processes may contribute to deposition on participant roofs, which in turn can be washed into water harvesting systems during rainfall events.

Another mechanism for increasing sulfate concentration around the home and in water harvesting systems is the combustion of fossils fuels containing sulfur (Perraud et al., 2015). The refining process of ores creates sulfur dioxide (SO_2_), among other byproducts, which in turn increases acid rain and acidic particle dry deposition, and potentially bioaerosols, bringing sulfates onto rooftops and into water harvesting devices (U.S. Environmental Protection Agency, 2020). Nearby facilities that would create SO_2_ exist in the participating communities of Hayden (i.e. ASARCO Smelter) and Miami (e.g. Freeport-McMoRan mine), AZ (Arizona Department of Environmental Quality, 2018). In urban areas, the combustion of fossil fuels is of greater concern, and SO_2_ concentrations are generally the result of vehicles, industrial facilities, and power generation from coal and to a lesser extent natural gas (U.S. Environmental Protection Agency, 2019).

### Comparison of SRB and coliform testing from lab categorized participants

Determining potable water quality is centralized around testing for indicator organisms, such as TC or *E. coli*. The reason for the historic use of these organisms is their presence in the gastrointestinal tracts and fecal waste of humans and animals (Ohrel & Register, 2006). Conversely, SRB were borne out of the need for a lower cost test, one which could be performed simply and without the need for a laboratory setting (Gupta et al., 2008). While SRB are naturally occurring in the environment, they are still commonly present in the gastrointestinal tract, and therefore a recognized sign that fecal contamination has occurred (Gupta et al., 2008).

Project Harvest Lab sample results revealed that when coliform bacteria were present in a harvested rainwater sample, SRB were determined to be present 15.1% (21/139 samples) of the time, while that percentage increases slightly when SRB are compared to samples positive for *E. coli*, at 20.0% (4/20 samples). However, the *E. coli*-SRB comparison set concluded that there was no association between the two organisms’ presences (*p*>0.05). Based on Spearman’s test, there were a few significant correlations with traditional TC MPN tests and the SRB MPN method. Spearman’s test and the chi-square test revealed a positive (*r*=0.245, *p*<0.05) correlation with TC MPN and SRB MPN, and a positive association (*r*=0.143, *p*<0.05) between TC presence/absence and SRB presence/absence, respectively. The point-biserial test also revealed a positive (*r*=0.197, *p*<0.05) correlation between TC MPN and SRB presence/absence.

Our data set does show a departure from literature, which generally displays moderate to strong correlations between TC and SRB. Khush et al. (2013), observed that when TC concentrations were intermediate to high (CFU≥1000/100 ml), SRB methods showed increasing sensitivity. In that experiment, the samples were collected from rural Southern India and compared presence/absence of SRB tests against the enumeration of TC (Khush et al., 2013). Another comparison of SRB tests and the Colilert® method in contaminated tap water in Indonesia determined that Colilert and SRB methods were qualitatively and quantitatively equal in their sensitivity of recovering their respective indicator organisms (Kromoredjo & Fujioka, 1991).

Overall, literature generally finds that SRB methods work for water quality testing (Table 8); however, the level of agreement among studies does vary (e.g. Sobsey & Pfaender, 2002). The aspects that most SRB studies agree on are that the tests are lower cost, have lower technological and training requirements, and have shorter time windows for results, and that SRB tests correlate well with traditional TC tests in environments where higher concentrations of fecal matter are of concern.

**Table 8:** A compilation of literature comparing H_2_S tests and coliform bacteria agreement

Water sample	Location	# of samples	Agreement	MPN range/100ml		Reference
Tap water	Lima, Peru	20	95%	<2	26	Ratto et al. (1989)
Well, rainwater, surface waters	Thailand	705	85%	<2	2400	Sivaborvorn (1988)
Surface and water distribution system	Chile	622	56%	N/A	N/A	Castillo et al. (1994)
Well water	India	1050	89%	<1	2400	Tambekar et al. (2008)
Harvested rainwater	Arizona, USA	226	46%	<1.0	>2419.6	Present study

The test method for enumerating SRB, total coliform, and fecal coliform vary across these studies. Data from Sobsey and Pfaender (2002)

### Participant preference for Lab or DIY methods

Since one of the goals of this research was to determine if SRB tests can function as an at-home low-cost and low-effort test, understanding participant ease and comfort with the method is important to consider when determining whether PathoScreen™ could be an effective alternative. Among those who indicated preference and rationale, Lab kits were primarily chosen (*n*=38, 60.3%) because they were easier to complete (*n*=7), less time consuming (*n*=4), and provided more contaminant concentration data, 33 contaminants vs. two (*n*=2). Of the 27% (*n*=17) who selected DIY, the flexibility in sampling and time frame (*n*=3) was the primary reason given. Finally, some participants asked to complete both kits as originally designed (*n*=8, 12.7%). Participants who selected both kits stated that they enjoyed conducting scientific activities (*n*=4) and were interested in receiving more harvested rainwater data (*n*=2).

Overall, the primary factor that influenced participant kit selection was time commitment and flexibility. The DIY method requires participation across 5 days, while the Lab method involves filling a bottle and dropping it off at the designated community location. Conversely, DIY participants had their results on day 5, while Lab participants received results at the end of the year during data sharing events. Less frequently mentioned was the ability to complete kits with family, allowing for the study to serve as an interactive tool and bonding experience.

### Study limitations

In general, the low number of samples positive for *E. coli* (20/229) in the overall sample set may have limited our ability to ascertain certain statistical trends. As previously stated, the initial study design intended for participants to complete both the DIY and Lab methods. However, based on participant and *promotora* feedback, it was decided to modify this approach, as the team recognized that having participants do both methods was not ideal and could be a burden to participants. An adjustment was made to utilize the participant Lab kit samples to perform both methods in the University lab. After delivery to the laboratory, samples were first processed for Colilert®, due to our obligation to report back lab-tested results to participants, and then turbidity, which required 10 ml. The remainder of the sample (100 ml) was then tested using the SRB method, which resulted in the exclusion of samples submitted with less than 210 ml of water.

**Fig. 5:**
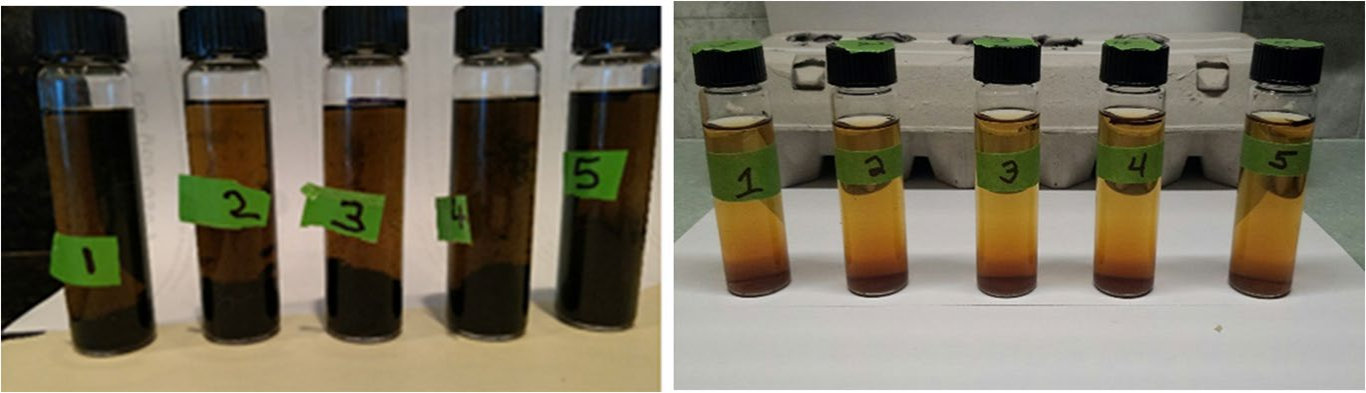
Examples of participant-submitted DIY results photos

Originally, we anticipated that participants would submit photos of their DIY results and this would serve as a mechanism for results validation. In total, only seven photos of DIY SRB vials were submitted with results, though the vast majority of DIY participants did submit their numeric results (an example of participant-submitted images can be seen in Fig. 5). While the seven submitted images are a small sample of the 229 submitted results for the DIY participants, of that group most participants (85%) were interpreting MPN results correctly and all interpreted presence/absence properly. Future studies should consider the target communities’ comfort with and access to technology, as well as technological literacy.

## Conclusion

Most SRB studies previously conducted were to determine fecal contamination in surface water (e.g. Sobsey & Pfaender, 2002). With regard to harvested rainwater, fecal contamination (from rodents, avian, and reptilian species) is one pathway, but not the sole pathway for contamination. Particles assimilated by falling rainwater, and eolian deposition of nearby mine tailings dust, pose potential supplementary sources for contamination in our partner communities. While the TC presence/absence test had a low level of agreement with the SRB presence/absence test (45.5%), there was an association between the two tests. Conversely, the *E. coli* presence/absence test had a high level of agreement (83.2%) with SRB tests, but no correlation, indicating that the agreement is likely owing to harvested rainwater samples lacking both *E. coli* and SRB.

The simplicity and safety of SRB tests (e.g. Hach’s Pathoscreen) do bode well for use by lightly trained personnel. However, early in Project Harvest, it became clear that internet access and technological literacy/comfort may have served as barriers to participation and access.

There are currently no standards for harvested rainwater and communities that make use of rainwater have water safety concerns. The SRB method could be used to screen rainwater quality for at-home use, specifically for those who use harvested rainwater for irrigation. The low sensitivity of SRB tests makes it difficult to say that SRB tests can unequivocally be suitable for harvested rainwater testing. The SRB method could, however, be recommended if certain conditions are met including (1) the person commonly notices animals on or around their roof/harvesting system, (2) cost is a barrier, and (3) and there is a lack of access to more advanced testing methods.

## Data Availability

The data sets generated during and/or analyzed during the current study are available from the corresponding author on reasonable request.
